# Physiological Mechanisms Underlying the Primary Respiratory Mechanism (PRM) and Cranial Rhythmic Impulse (CRI) in Osteopathy: A Systematic Review

**DOI:** 10.3390/healthcare12242503

**Published:** 2024-12-11

**Authors:** François Mériaux, Laurent Stubbe, Alice Guyon

**Affiliations:** 1 Animal Osteopath, 33 Rue Colonel Bougault, 38100 Grenoble, France; fm@osteo-animalier.com; 2 ESO-Paris Recherche, Ecole Supérieure d’Ostéopathie—Paris, F-77420 Champs Sur Marne, France; laurent.stubbe@eso-suposteo.fr; 3 CIAMS EA 4532, Université Paris-Saclay, F-91405 Orsay, France; 4 CIAMS EA 4532, Université d’Orléans, F-45067 Orléans, France; 5 Faculté des Sciences, CRPN UMR 7077-Aix Marseille Université, Campus St Charles, CNRS-3, Place Victor-Hugo, F-13331 Marseille Cedex 3, France

**Keywords:** cranial rhythmic impulse, primary respiratory mechanism, cranial osteopathy, osteopathy in the cranial field, cranio-sacral therapy, osteopathic cranial manipulation

## Abstract

Background: Cranial Rhythmic Impulse (CRI) or Primary Respiratory Mechanism (PRM), movement felt on the scalp or the rest of the body, respectively, is a fundamental concept used by osteopaths in their practice for their diagnosis and treatment. However, the physiological basis of this phenomenon remains unclear. Sutherland, the founder of cranial osteopathy, proposed in 1939 that PRM was due to the movement of the cranial bones pulled by the meninges, themselves pushed by the fluctuation of cerebrospinal fluid and the motility of the central nervous system. Since then, Sutherland’s theory has become dogma, despite scientific progress refuting it, and few osteopaths have attempted to find better explanations. Objective: This systematic review of Medline, Science Direct and the Cochrane Library indexed electronic databases explores current knowledge of the physiological mechanisms underlying the Primary Respiratory Movement (PRM) or Cranial Rhythmic Impulse (CRI). Methods: We entered the following identified keywords: “osteopathy in the cranial field”; “cranial rhythmic impulse”; and “primary respiratory mechanism”. We identified 193 studies, evaluated 115, and identified 28 articles that fulfilled our criteria. We classified the studies in terms of methodological rigor, types of studies and tools used. No study had three good-level items, and only five studies had two good-level items corresponding to the type of study and tools used. The protocol of the review has been registered on PROSPERO-CRD42023488497. Results: Out of the 28 articles, 20 referenced at least one of Sutherland’s hypotheses, often quoting the model to critique or challenge it, while 25 of them refer to other hypotheses and/or mechanisms underlying PRM/CRI: 11 concern vasomotion in blood vessels (7) and lymphatic vessels (6), 20 THM waves, 14 heart rate variability, 9 ventilation rate, 2 the extra-cellular matrix and 1 oxidative metabolism. Conclusions: Although Sutherland’s theory remains prevalent in general beliefs, in scientific literature, THM waves driven by autonomous system activity have gained prominence, emerging as the leading hypothesis. The results from this systematic review confirm the need for a paradigm shift for the CRI/PRM in osteopathy, and for more rigorous evaluation and communication on a model in step with evolving scientific data.

## 1. Introduction

Primary Respiratory Mechanism or Movement (PRM) or Cranial rhythmic impulse (CRI), the same movement felt on the cranium [1], is a fundamental concept used by osteopaths in their practices, which contributes to their diagnosis and treatment. Historically, it was discovered by the osteopath William Garner Sutherland (1873–1954) [1]. While observing the sutures of the skull, he had the intuition that they were made for movement, and studied it for 40 years before publishing his findings “The Cranial Bowl” [2]. This model is based on five principles: the inherent mobility of the central nervous system and the spinal cord; the fluctuation of the cerebrospinal fluid (CSF); the reciprocal tension membranes; the articular mobility of the bones of the skull; the involuntary movement of the sacrum between the iliac bones [2].

According to his cranial model, it would be possible for a trained practitioner to palpate and enhance the presumed movements of each cranial bone when needed using both hands on the skull. Restoring this mobility would have a positive effect on the entire body, enhancing homeostasis and health. The PRM/CRI rhythm is regularly used as a diagnostic tool by osteopaths. Applied to patients, this model seems to work and has helped millions of people worldwide.

Some rhythms with similar rates to PRM/CRI can also be obtained by instrumentation, which could objectify its existence [3]. In a recent study, Rasmussen and Meulengracht [4] used a machine they developed to measure directly physical rhythmic movements on the head. They found that healthy humans (n = 50) have a rhythm around the head, with a mean of 6–16 cpm and a narrow normative range (4.25–7.07). Nelson [5,6] demonstrated a statistically significant correlation between cyclic changes in blood flow measurements and the CRI, and concluded later [6,7] that ‘cranial’ manipulation had a significant effect on blood flow. More recently, Pelz [8] confirmed that skin blood flow exhibits parallels with the PRM/CRI using photoplethysmography (PPG) measurements and showed that osteopathic manipulative treatment could improve coordination or synchronization effects.

Considering that we have enough studies to believe that PRM/CRI could exist and that a trained practitioner could feel and influence it, it is now legitimate to wonder about the origin of these micro-movements.

Literature data on physiological mechanisms supposed to underlie the PRM/CRI rhythm are scarce. Several authors questioned the physiological mechanisms underlying the PRM/CRI, but to our knowledge, the latest review on this topic dates from 2003 [3]. Since then, the fact that science progressed on these subjects, documenting diverse rhythms driven by the autonomic nervous system, has prompted us to perform a systematic review of the literature on this topic in order to decipher the various hypotheses of the physiological mechanisms supposed to underlie the PRM/CRI rhythm. The initial model proposed by Sutherland is no longer consistent with current knowledge of physiology, highlighting the need for a paradigm shift and for more rigorous evaluation and communication of a model that is in line with the evolution of scientific data. To this effect, we performed a structured systematic review on this subject.

## 2. Materials and Methods

### 2.1. Study Design

This study is a systematic review based on the following research question established using the PICO approach: “What are the different hypotheses about the physiological mechanisms thought to underlie the primary respiratory mechanism or movement (PRM) or the rhythm of the cranial rhythmic impulse (CRI)?”.

We conducted a systematic literature search during October 2023 in the indexed electronic databases MEDLINE, ScienceDirect and Cochrane Library. Searches were conducted without date restriction. Only complete studies in English describing and/or seeking to clarify the physiological mechanisms of PRM or CRI were included. The criteria used for the analysis of the studies were the inherent motility of the central nervous system and spinal cord; the fluctuation of CSF; the motility of intracranial and intraspinal membranes; and the so-called “joint mobility” of the cranial bones. Three reviewers evaluated the studies independently and resolved any disagreements by consensus.

The protocol of the review has been registered on PROSPERO-CRD42023488497.

**Figure 1:**
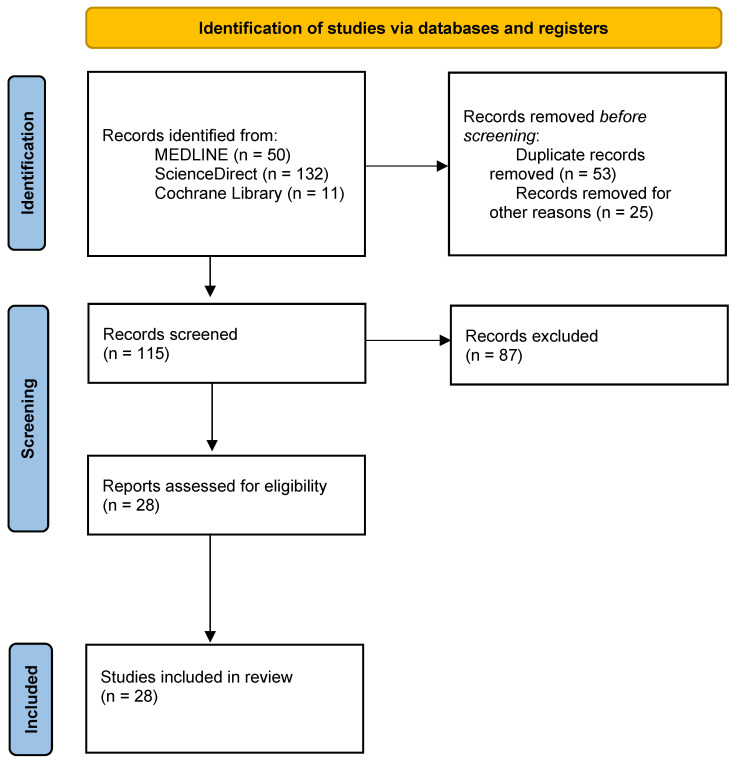
Flow chart indicating the different steps followed for the article selection for the systematic review.

The methodology was the following (Figure 1):

### 2.2. Search Strategy

We identified keywords describing and/or seeking to explain the physiological mechanisms of PRM or CRI that were entered into each of the electronic databases MEDLINE, ScienceDirect and Cochrane Library: “osteopathy in the cranial field”; “cranial rhythmic impulse”; and “primary respiratory mechanism”.

The search was conducted during October 2023, with no filters for search criteria.

### 2.3. Systematic Selection Process

We excluded duplicate articles in databases, articles that were not in English, those that were not available in full text, and inappropriate studies that did not have clear information regarding the description and/or explicitness of the physiological mechanisms of PRM or CRI.

Study titles and abstracts were analyzed and studies that did not meet the eligibility criteria were excluded. The full texts of all identified studies were analyzed and the eligibility criteria were again applied. In each article selected, we retained the elements that were relevant to the PRM/CRI and cited them in the text.

### 2.4. Evaluation Criteria

As the articles selected covered a wide spectrum of study types, the grids usually used to evaluate the clinical literature were unsuitable. A grid of criteria (Table 1) to assess the relevance of the review, the robustness of the type of study/publication and the precision, reliability and reproducibility of the PRM/CRI measurement tools was created according to criteria established in the literature with the consensus of the authors but was not subjected to an independent validation process.

To evaluate relevance of the journal, we calculated the sum of Journal type + Journal quartile (Q) + Impact Factor (IF), with the following arbitrary values: 

Journal type: Professional journal → 0; Non-professional journal in the field of complementary and alternative medicines →1; General interest scientific journal → 2,

Journal quartile (Q): Q N/A → 0; Q > 1 → 1; Q =1 →2, 

Impact factor (IF): IF N/A → 0; IF≤ 2 → 1; IF >2 → 2. 

The colors correspond to the level of relevance, robustness and accuracy of the studies: red (very low), orange (low), yellow (medium), green (high level).

## 3. Results

**Figure 2:**
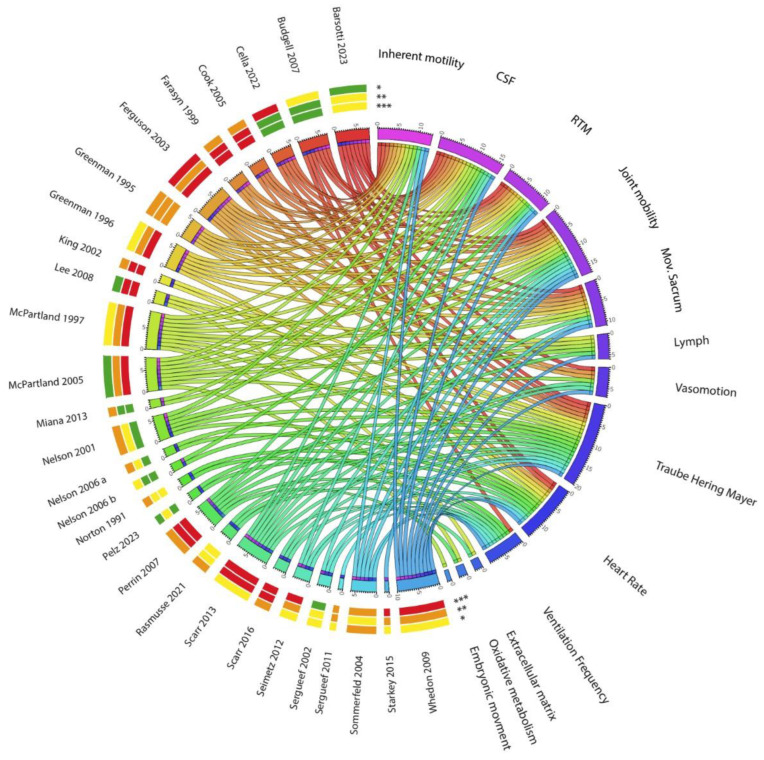
Circos chart showing the main topics covered in the articles. On the left of the circle are the 28 articles selected in the systematic review with the indication of relevance levels of the journals (*), the robustness of the PRM/CRI evaluation level across studies (**) and the accuracy/reliability/reproducibility of the PRM/CRI measurement tools level (***) indicated by colors (0 = red, 1–2 = orange, 3–4 = yellow, 5–6 = green), as determined by the grid of criteria indicated in Table 2. On the right appear the different topics covered in the articles, each topic being also represented by a color [9].

In the search, we identified 193 studies. We evaluated 115 studies, and among them, we excluded 87 (Figure 1, Table 1). Of the 28 articles identified (Table 2) and Circos [9] (Figure 2), 18 articles were identified in PubMed Central, 16 in ScienceDirect and 2 in the Cochrane Library. Twenty articles were identified using the keyword “cranial rhythmic impulse”, 17 with “primary respiratory mechanism” and 11 with “osteopathy in the cranial field”. Eight are theoretical models or hypotheses, four narrative reviews, three reviews, one note, three pilot studies and nine clinical studies. Of the 28 articles, only 4 were published in journals rated at a good level, 10 in medium-level journals and the remainder at a low or questionable level: 6 are from J Am Osteopath Assoc, 3 Altern Ther Health Med, 4 International of Osteopathic Medicine, 2 J Osteopath Med, 2 Journal of Manipulative and Physiological Therapeutics, 1 Man Ther, 1 Physical Medicine and Rehabilitation Clinics of North America, 5 J Bodyw Mov Ther, 2 Explore, 1 Healthcare and 1 Scientific Reports. Four studies out of 28 have a “type of study” evaluated as a good level, 8 at an average level and the others at a low or debatable level. Regarding the tools used, 8 out of 28 items were assessed as good, 3 as average, 3 as poor and 14 as questionable. No study had 3 good-level items, and only 5 studies had 2 good-level items corresponding to the type of study and tools used. Fifteen of the 28 studies have one or two items classified as questionable.

For each article, the table summarizes the journal in which it was published, the type of article, the tools used, the main findings and the article’s keywords. The colors represent the degree of quality of the journal, the type of article and the tools used, according to the criteria we have defined in Table 1: red (very low), orange (low), yellow (medium), green (high level).

The tools and methods for objectification of PRM used in these articles were: anatomic material, laser Doppler, electroencephalogram, electrocardiogram, spirometer, blood pressure, 2 servo-activators positioned on the temporal mastoids and osteopathic palpation.

Of the 28 validated articles (see Table 1), 20 refer to at least one pillar of Sutherland’s hypotheses (see Table 1). Of these, 13 mention cerebral spinal mobility, 16 mention CSF fluctuation, 12 mention reciprocal tension membrane mobility, 17 mention cranial bone mobility and 11 mention involuntary movement of the sacrum between the iliac bones.

In 25 of the 28 articles, other hypotheses and/or mechanisms underlying PRM/CRI are put forward and/or studied: 11 concern vasomotion (7 of blood vessels and 6 of lymphatic vessels), 20 concern THM waves, 14 concern heart rate variability (HRV), 9 concern ventilation rates, 2 concern the extra-cellular matrix, 2 concern oxidative metabolism and 1 concerns embryology.

### 3.1. Sutherland’s Five Theory Principles

As mentioned by McPartland [10], Sutherland is the one who discovered the PRM. Of the 28 selected articles, 20 mention at least one pillar of Sutherland’s theories (Figure 2). Greenman and McPartland [11] insisted on the fact that “The pioneering work of Sutherland included years of research into the anatomy of the skull, clinical observation of skull mobility in normal asymptomatic patients, and abnormal cranial mobility in patients with a variety of symptoms”.

Barsotti [12], Ferguson [3], Greenman [11,13], McPartland [10,14], Scarr [15] and Whedon and Glassey [16] present Sutherland’s theories and its five pillars in their introductions.

The five pillars are detailed below with selected articles that mention them:

#### 3.1.1. The Inherent Motility of the Central Nervous System and Spinal Cord

The inherent motility of the central nervous system and spinal cord is mentioned in 13 articles (Figure 2) [3,5,10,11,12,13,14,15,16,17,18,19,20]. It is evoked by Sommerfeld [20] (“Several physiological models try to explain the PRM such as the hypothesis of cerebral motility (Sutherland, 1998),…”).

#### 3.1.2. The Fluctuation of the CSF 

The fluctuation of the CSF is mentioned in 16 articles (Figure 2) [3,5,10,11,12,13,14,15,16,17,18,19,21,22,23,24] and described by Farasyn [19] (“He named this motion the ‘primary respiratory mechanism’ and suggested that rhythmical brain movement might be responsible for cerebral ventricular and CSF fluctuations”.), but also by McPartland [14] (“Initially, Sutherland [2] proposed that pulsations arise from rhythmical motions of the brain, causing dilatation and contraction of cerebral ventricles, generating a pulse wave of CSF”.). Scarr [15] also developed this point (“Sutherland’s suggestion of inherent expansion and contraction of brain tissue was followed by a hydrostatic model, where fluctuations in the production and re-absorption of CSF cause periodic changes in intracranial volume, articular mobility of cranial bones and sacral mobility through meningeal connections”).

#### 3.1.3. The Motility of the Intracranial and Intraspinal Membranes 

The motility of the intracranial and intraspinal membranes is mentioned in 12 articles (Figure 2) [3,6,10,11,12,13,14,15,16,17,20,21]. For example, Greenman [13] says “Alteration in tension from side to side, front to back, or top to bottom can influence the mobility characteristics of the osseous elements of the cranium. The RTM plays an important role in normal venous drainage”.

Similarly, Ferguson [3] claims “[…] the dural membranes. These stabilize the spherical cranium three-dimensionally to stop it from over-expanding in any direction. These are in a state of reciprocal tension so that the cranium tends to alternately expand laterally or sagittal direction, which seems to be confirmed by experimental evidence”.

Finally, Cook [21] also describes: “Tensegrity principles state that bones transmit compressive forces, whereas membranes transmit tensile forces. Hence, the bony skull accommodates external pressures and internal compressive stresses by “bottoming out” on its sutural surfaces […] The tentorium is stretched by lateral expansion of the parietals, and this tension pulls the occiput forward, compressing the occipitoparietal sutures, with the effect of expanding the parietals. This reciprocal and synergistic relationship between tension and compression is characteristic of a tensegrity structure”.

#### 3.1.4. Joint Mobility of Cranial Bones 

Joint mobility of cranial bones is mentioned in 17 articles (Figure 2) [3,7,10,11,12,13,14,15,16,17,18,20,21,22,23,24,25] and described by Cook [21]: “It is over 80 years since Sutherland discovered the motion of cranial bones by inspection of a disarticulated skull and the application of the “form follows function and function follows form” principle to sutures. The original concepts of sphenobasilar synchondrosis (SBS) motion continue to be central to basic techniques in Craniosacral Therapy and Cranial Osteopathy (CST/ CO), as does the concept of non-bony sutures, to accommodate this model of cranial motion”. Seimetz [24] also wrote “Practitioners of CO believe that rhythmic motion of the cranial bones exists due to fluctuations of the cerebrospinal fluid pressure and arterial blood pressure”.

Miana [22] said that “Cranial manipulation has received criticism because of the subtle, difficult to learn techniques, and controversy over whether or not cranial bone structures move in accordance the theory of the PRM postulated by Sutherland”. Finally, Starkey [25] mentioned that “Sutherland and most subsequent craniosacral authors hold that, in adults, cranial motion is, in part, enabled by the movement of the synostosis”.

#### 3.1.5. The Involuntary Movement of the Sacrum Between the Iliac Bones, Also Named the “Core Link” Hypothesis 

This hypothesis is mentioned in 11 articles (Figure 2) [3,10,11,12,13,14,15,16,18,19,20]. It is rejected by Farasyn [19], who claims that there is no synchronicity between the occiput and sacrum as described in the Sutherland model and proposes another model.

Only 8 articles out of 28 (Figure 2) do not mention any of the five pillars of Sutherland’s theory. In 25 of the 28 articles (Figure 2), other hypotheses and/or mechanisms underlying PRM/CRI are put forward and/or studied as described below.

### 3.2. Vasomotion or Vasomotricity

One hypothesis that emerges in 11 out of those 28 articles is that the PRM could be secondary to vasodilatation of the blood vessels (7) or lymphatic vessels (6) (Figure 2) [3,5,10,12,14,15,16,19,23,26,27].

Christ [28] measured cyclic changes in limb volume within the CRI range and suggested that they were due to variations in arterial pressure, arteriolar vasomotion and possibly lymphatic diameter.

Greenman [13] states that “Other intracranial oscillators include cerebral blood volume and cortical oxidative metabolism, which fluctuate at 9 cpm. Cerebral blood velocity also has an oscillation independent of systemic circulation”.

McPartland [10] outlines that “Fluid pressure within lymphatic vessels also oscillates rhythmically, between 7.5 and 10 cpm, asynchronously with diaphragmatic respiration”.

Farasyn [19] proposes that the intrinsic movement of cranial bones, fascia and organs may be caused by local venomotion pulsation.

Ferguson [3] pretends that venous pressure is too low to explain the CRI. However, he says that “Like arteries, the larger lymph channels exhibit pulsatile dilatation and constriction”. However, he does not think that lymphatic vessels play a role in CRI, as there are no large lymph vessels within the cranium. In contrast, he outlines evidence that arterial vasomotion occurs at rates commonly associated with CRI. He suggests that is possible that individual practitioners focus on different rates of vasomotion generated by different-sized arteries.

Finally, Perrin [23] argues that the CRI is the rhythm produced by a combination of pulsations of central lymphatic drainage induced by the SNS and CSF drainage from the neuraxis.

### 3.3. Autonomic Nervous System

Most investigators and practicing clinicians recognize a strong link between the CRI and the autonomic nervous system [28], see (Figure 2).

Historically, in 1865, Traube measured an oscillation in the periphery, which was attributed to intrathoracic pressure fluctuation of pulmonary respiration and was noted to persist after the cessation of respiratory motion. Hering independently demonstrated Traube’s discovery. Later, Mayer identified an additional, lower-rate oscillation [29].

Recordings of both variability in heart rate and Traube–Hering modulation resemble the rate and rhythm of the CRI as recorded by Frymann [30], although CRI-like oscillations in cerebral blood velocity have also been recorded, independent of systemic circulatory parameters. These oscillations may be linked to changes in cerebral blood volume and oscillations in cortical and oxidative metabolism, which occur at a rate of 9 cpm [10].

#### 3.3.1. THM Waves

Twenty articles dealt with the fact that the cranial rhythmic impulse is synchronous with the THM oscillation (Figure 2) [3,4,5,6,7,8,10,12,14,15,16,17,18,21,23,26,27,29,31,32].

Several authors relate that arterial blood pressure does fluctuate rhythmically within the range ascribed to the cranial rhythm [3,10,16,26,27,31].

Measurements of arterial blood pressure by many researchers show two peaks, at 0.25 Hz (HF) and at 0.1 Hz (LF). These frequencies are 15 cpm and 6 cpm, respectively. The HF peak is generally linked to respiration and depends on autonomic function [10], mainly parasympathetic activity [17]. The LF peak has been identified and attributed to an intrinsic slow rhythm in sympathetic output [17] and would be linked to input from baroreceptors and chemoreceptors in the carotid sinus and aortic arch [3].

Protocols to simultaneously measure both CRI and THM, as the one described by Nelson [5], allowed for the demonstration of a correspondence between the two rhythms. These authors demonstrated a 1:2 ratio between the cranial rhythmic impulse palpated by physicians and the THM wave, and were even able to ascribe certain aspects of THM oscillations with the so-called fast tide of between 8 and 12 cpm and the slow tide rate of 0.6 cpm [29].

Nelson [6,31] later demonstrated a statistically significant correlation between cyclic changes in blood flow measurements and the CRI. In their subjects, the authors measured a frequency of THM oscillation of 6.75 ± 4.50 cpm and a measured CRI of 4.54 ± 2.08 cpm. The authors concluded that the findings imply that the PRM and the THM oscillations are simultaneous, if not the same phenomenon.

Whedon [16] also claimed that given the CRI and the THM oscillation are both whole-body phenomena that occur simultaneously, it seems likely that the two are actually the same.

Scarr [15,27], in the current anatomical knowledge of his time and unable to explain the THM waves, proposed that cyclic changes in vascular volume would alter the tension in associated myofascia and create patterns of motion that are palpable. Regular oscillations of arterial pressure and volume would then likely influence the behavior of the tissues surrounding them.

It has been demonstrated that manual cranial techniques affect THM [18,23]. Sergueef [7] mentions increases in the amplitude of the ‘Traube–Hering’ wave (this may have been incorrectly named and was most likely the Mayer wave) following cranial manipulation in a small trial using 23 subjects, which is also reported in four other articles [3,23,26,31].

A later study by Nelson [5] concluded that ‘cranial’ manipulation had a significant effect on blood flow, but that practitioners can recognize a variety of physiological frequencies, which may have contributed to earlier confusion regarding the precise CRI rate. Variations in vessel caliber might influence limb volume and relate to the palpable CRI. Rhythmic changes in fluid volume might be responsible for the sense of limb expansion and contraction observed by practitioners [15].

More recently, Pelz [8] claimed that “Previous studies on CRI/PRM dynamics have identified such oscillations as (TH or THM; respiration) waves with a frequency centered at approx. 0.1 Hz. Technical approaches investigating the physiology underlying the CRI were promising yet inconclusive due to their poor match with palpation. Association of CRI/PRM with TH waves in blood pressure, heart rate, respiration, and skin blood flow have been suggested as physiological correlate for the palpation of the CRI/PRM within all parts of the body. However, palpated CRI rates differed frequently by a factor 2 from rates recorded using technical instrumentation. The CRI/PRM thus remains an enigmatic phenomenon”.

**Table 2:** Evaluation of the relevance of selected elements of the publications.

First Author, Year, [Ref.]	Revue		Article Type		Tool		Key Words, Main Findings
Barsotti 2023 [12]	Healthcare		Hypothesis		Perspective article		Explored several key aspects related to craniofacial tactile mechanosensation and its implications in exo- and endocranial communication.
Budgell 2007 [17]	Journal of Manipulative and Physiological Therapeutics		Hypothesis		A large part of the analysis based on a simple game with a disarticulated skull		Joint mobility of the cranial bones Overview of Sutherland’s bone theories
Cella 2022 [18]	J Osteopath Med.		Hypothesis		Study of the literature (anatomy, physiology, osteopathy) and palpatory obsevation research on subjects		Fluctuation of cerebrospinal fluid (CSF) Intrinsic skull movement may be caused by local venous pulsation
Cook 2005 [21]	Journal of Bodywork and Movement Therapies		Narative Review		Review of current research into the physiological aspects behind the theories and practice of cranial osteopathy		Overview of Sutherland’s theories and his five pillars Arterial vasomotion occurs at rates commonly associated with CRI Role of the sympathetic nervous system (SNS) in its control of vasomotion
Farasyn 1999 [19]	Journal of Bodywork and Movement Therapies		Clinical trials 55 subjects 1978–1992		Cranial and sacral findings were recorded on an examination form illustrated in a previous report. Palpation and clinical study.		Overview of Sutherland’s theories and his five pillars
Ferguson 2003 [3]	Journal of Osteopathic Medicine		Narative Review + Clinical cases		Study of cranial osteopathic literature compared with the results of a case study		Overview of Sutherland’s theories and his five pillars Motor source of the CRI may be extracranial (skin tissue, muscles, etc.) Close link between the CRI and the autonomic nervous system
Greenman 1995 [11]	J Am Osteopath Assoc.		Theorical model		Literature review (anatomy, physiology, osteopathy)		Overview of Sutherland’s theories and his five pillars PRM is linked to calcium waves and accompanying water, and is associated with alterations in the viscosity and charge of the extracellular matrix.
Greenman 1996 [13]	Physical Medicine and Rehabilitation Clinics of North America		Narative Review		Literature review (anatomy, physiology, osteopathy)		Introducing Sutherland as the discoverer of PRM Fluid pressure in lymphatic vessels also oscillates rhythmically, asynchronously with diaphragmatic breathing Changes in cerebral blood volume and oscillations in cortical and oxidative metabolism, heart rate variability, Traube-Hering modulation…Autonomic nervous system dysfunction may modify training frequency in PRM
King 2002 [29]	J Am Osteopath Assoc.		Narative Review		Literature review (anatomy, physiology, osteopathy)		General presentation of Sutherland’s theories and his five pillars moving towards the biodynamic embriological model of cranial osteopathy
Lee 2008 [26]	Explore		Pilot study 10 healthy subjects		20-channel EEG system Braintech-3000. Compression of the fourth ventricle on 10 healthy subjects		Compression of the lateral occipital bone impacts the cerebellar tent, modifying the pressure inside the fourth ventricle and subsequently the entire cerebrospinal fluid pressure.
McPartland 1997 [10]	Altern Ther Health Med.		Model		Literature review (comparative anatomy, physiology, osteopathy)		Overview of Sutherland’s theories and his five pillars Fluctuations in cerebrospinal fluid (CSF) production and reabsorption Variations in vessel caliber (vasomotion) can influence limb volume and be linked to palpable CRI TraubeeHeringeMayer oscillations in the vasculature also provide a pathway for CRI to reach the limbs The fascial network and the extracellular matrix continuous with it and surrounding virtually every cell in the body could be part of the PRM.
McPartland 2005 [14]	Explore		Review		Literature review (anatomy, physiology, osteopathy)		Joint mobility and cranial bone deformity
Miana 2013 [22]	Journal of Bodywork and Movement Therapies		Research article 49 healthy subjects/two operators		Palpatory findings of 2 examiners recorded via two foot switches.		Mobility within the bony and membranous structures of the skull, cranial mobility hypothesis An autonomous rhythmic phenomenon inherent to all living organisms, independent of thoracic respiration and cardiac pulse
Nelson 2001 [5]	J Am Osteopath Assoc.		Review		Review of the relevant literature and an examination of a number of adult cranial bases, medial sections of the skull and sphenoid bones		Study of cranial bone joint motility (sphenobasilar synostosis)
Nelson 2006 a [31]	J Am Osteopath Assoc.		Review		Literature review (anatomy, physiology, osteopathy)		Sutherland model Motility of intracranial and intraspinal membranes Correspondence between CRI and THM waves Physiological factors affecting normal CSF circulation include cardiovascular (HRV), respiratory and vasomotor (vasomotion) influences. The balance between the sympathetic and parasympathetic nervous systems can result in the frequency entrainment of several biological oscillators
Nelson 2006 b [6]	Journal of Manipulative and Physiological Therapeutics		Research article 8 urethane-anesthetized adult male Wistar rats		ECG, BP, CSF pressure, ventilatory flow, and chest excursion were recorded on a computer		CSF pressure oscillations are driven and entrained by ventilation Pulsed arterial pressure contributes little to CSF pressure oscillations
Norton 1991 [33]	J Am Osteopath Assoc.		Research article 40 healthy subjects		Brain alpha-band power (ABP) electroencephalogram (EEG)		Study of the neurophysiological connection between the occiput and the sacrum
Pelz 2023 [8]	Scientific reports		Pilot study		Using a standard Osteopathy in the cranial field procedure, cranial vault, two experts palpated and digitally marked CRI frequencies in 25 adults		Autonomic nervous system (ANS) activity in the low frequency (LF) and IM bands in photoplethysmographic (PPG) recordings of the forehead skin was probed with the momentary frequency of highest amplitude (MFHA) and spectrum wavelet amplitude (WAS) in examiners and participants.
Perrin 2007 [23]	J Am Osteopath Assoc.		Note		Review of osteopathic cranial literature		Results and implications of the THM relationship with the primary respiratory mechanism Possible effects of osteopathy in the cranial field on heart rate variability
Rasmusse 2021 [4]	J Bodyw Mov Ther.		Pilot study 12 subjects		Laser-Doppler flowmetry vs. Palpation Perfusion monitor (Transonic Systems Inc, Ithaca, NY)		Statistical comparisons have demonstrated a relationship between the palpated cranial rhythmic pulse and the THM wave
Scarr 2013 [15]	International Journal of Osteopathic Medicine		Research article 44 ostéopathes 44 subjects		Palpation of the CRI by osteopathic physicians skilled in the methods of cranial osteopathy was compared with simultaneously recorded laser-Doppler flowmetry.		Doctors tend to palpate the cranial rhythmic impulse and TraubeHering oscillation in a 1:2 ratio
Scarr 2016 [27]	J Bodyw Mov Ther.		Research article 26 subjects 28 ostéopathes		Compression of the 4th ventricle and laser transcutaneous blood flowmeter (Transonic Laser-Doppler Monitors, BLF21 Series, Transonic Systems, Inc, Ithaca, NY).		Compression of the fourth ventricle (CV-4) is a non-invasive manual procedure that affects the cranial rhythmic impulse, a phenomenon recognized by practitioners as concomitant with Traube-Hering (TH) oscillations
Seimetz 2012 [24]	International Journal of Osteopathic Medicine		Computer model		A computer model has been developed to determine the deformation force produced by tissue pressures on the cutaneous mechanoreceptors of an examiner’s hand in the presence of rhythmically changing arterial and venous pressures in the skin of the examiner and subject.		CRI is associated with slow adaptation of the skin mechanoreceptors of both patient and practitioner, and the sources of change in these tissue pressures are the combined respiratory and cardiovascular rhythms of the examiner and subject.
Sergueef 2002 [7]	Altern Ther Health Med.		Hypothetical Model		Review of literature, animal studies and clinical outcomes in patients with chronic fatigue syndrome		The CRI is the rhythm produced by a combination of central lymphatic drainage pulses induced by the sympathetic nervous system and cerebrospinal fluid drainage from the neuraxis
Sergueef 2011 [32]	International Journal of Osteopathic Medicine		Research article 50 subjects		Two servo actuators CAL 12-010-5 (SMAC Corporation 5807 Van Allen Way Carlsbad, California, USA 760-929-7575), having a sensitivity to detect physical movements of 1 mm. were positioned on the skin at the positions of the temporal mastoids, maintaining a persistent contact of 10 g at all measurement times		A rhythm distinct from the arterial and respiratory rhythms has been distinguished at all times
Sommerfeld 2004 [20]	The Journal of Manual & Manipulative Therapy		Model		Literature review (anatomy, physiology, osteopathy)		Correspondence between CRI and THM waves. Cyclic contraction of these fascial tubes in synchrony with changes in vascular volume (both intra- and extra-muscular) could influence in a particular way the configurations of the crossed helical fibers within their walls
Starkey 2015 [25]	International Journal of Osteopathic Medicine		Research article 23 subjects		Blod flow velocity recordings with a Laser Doppler flowmetry probe placed on the left earlobe of each subject. After a 5 min baseline, cranial manipulation, consisting of equilibration of the global cranial motion pattern.		Manual cranial techniques affect the THM waves
Whedon 2009 [16]	Altern Ther Health Med.		Research article 734 subjects		CRI palpation		Students taking part in the academic program were taught to palpate the CRI and were tested to establish their ability to monitor it. The data in this study are drawn from these assessments, half the group being examiners and the other half subjects, and then the roles were reversed.

#### 3.3.2. Heart Rate Variability (HRV)

Fourteen articles mentioned that the CRI/PRM could be linked to the HRV (Figure 2) [3,4,5,8,10,11,12,13,16,17,20,24,29,31,33]. Ferguson [3] and Greeman [13] explain that variations in blood pressure correspond to variations in heart rate. The HRV or heart period can be measured from the spectral analysis of sequences of R-R intervals from electrocardiograms. The power spectral densities show a predominance of certain frequencies, which show the same two peaks as the blood pressure at about 0.25 Hz and 0.1 Hz, as also mentioned by Sommerfield [20].

Greenman explains that the high-frequency component HF is associated primarily with respiratory sinus arrhythmia and is attributed primarily to parasympathetic action. The low-frequency component LF is almost entirely accounted for by a baroreflex mechanism and is to some degree an index of sympathetic activity (Nelson) [31].

Greenman remarks that the different autonomic neural components become balanced, and HRV has been shown to assume a rhythmic sinusoidal pattern. This pattern resembles the CRI tracings that have been obtained in other studies. The blending of these high-frequency and low-frequency components would occur at approximately 0.20 Hz, or around 12 cycles per minute, close to known CRI rates [13].

Greenman [11] explains that the chronic loss of HRV is linked with aging, diseases of the autonomic nervous system, diabetes, increased cardiac mortality, and psychological disorders including depression and panic attacks. Conversely, Whedon [16] and Nelson [31] note that “THM waves are mediated by the autonomic nervous system and along with increased HRV, are considered to be markers of good autonomic balance”.

King [29] mentions studies showing that a respiratory rate of 0.1Hz produced a marked effect on synchronization and increased variability in all cardiovascular rhythms, manifested at both the human nervous and vascular systems levels, with favorable effects on cardiovascular events. Whedon [16] also gives several examples suggesting that osteopathic care may have profound effects on autonomic balance.

### 3.4. Ventilation Frequency

In nine of the selected articles (Figure 2) [4,7,16,17,20,23,24,27,33], breathing and/or ventilation are mentioned in the mechanisms associated with or to be dissociated from the PRM/CRI. Scarr and Sergueef [7,27] examined the impact of the ventilatory rate on the PRM via the ANS, focusing on blood pressure variations that reflect sinus respiratory arrhythmia. Budgell and Whedon [16,17] studied the influence of ventilation on CSF pressure, showing a correlation between CSF pressure oscillations and ventilatory rate. Whedon [16] also observed that normal breathing can induce periodic brainstem movement, resulting in low-frequency brainstem oscillation. In the study by Perrin [23], the source of the CRI could be induced by the influence of ventilation and pulse on the thoracic (lymphatic) duct pump. Seimetz [24] did not associate ventilation or PRM/CRI, but referred to the results of a study indicating that lateral biparietal compression could affect the ventilatory and cardiac rate reversibly upon cessation of compression. Norton and Sommerfeld [20,33] studied the interaction between subject and/or observer ventilation on the PRM and CRI, noting that the rhythms were related to the activation of slowly adapting skin mechanoreceptors by examiner and subject tissue pressures, and that changes in these tissue pressures were due to the combined respiratory and cardiovascular rhythms of the examiner and the subject. More recently, Rasmussen [4] observed a third rhythm distinct from the ventilatory and cardiac rates in all the subjects of his study.

### 3.5. Microcirculation, Fascial Network and the Extracellular Matrix

Two publications investigate the hypothesis of a link between PRM, microcirculation, fascial network and the extracellular matrix (Figure 2) [15,26].

The model of the PRM presented by Lee [26] explains tissue expansion/contraction by describing the pulsatile activities of cells and the extracellular matrix that surrounds them. The exchange between the nutrient capillaries and cells, on one hand, and between the cells and lymphatics, on the other hand, would be involved. He states, “It is my belief that what we feel in the tissues as the tide is related to the waves of calcium ions and the accompanying water, which is associated with alterations in the viscosity and charge of the matrix”.

Scarr [15] explains that both the fascial network and the extracellular matrix surrounding virtually every cell in the body could both be part of the PRM. The extracellular matrix also connects and transfers tension through transmembrane proteins to an internal cellular lattice called the cytoskeleton. Changes in cytoskeletal tension cause enzymes and substrates situated on the lattice to alter their activity, influencing cellular respiration and metabolism, and ultimately switching between different functional states such as growth, differentiation or apoptosis. As oscillations are a common feature in human physiology, a cyclic transfer of tension into the cytoskeleton may also enhance cellular metabolism, and be amenable to therapeutic intervention through the cranial and fascial parts of the PRM.

### 3.6. Metabolic Hypothesis

In their articles, McPartland and Perrin (Figure 2) [10,23] mention that the cortical oxidative metabolism might be part of the oscillators that can be transduced into tissue movement and be part of the CRI.

### 3.7. Embryology

Jealous developed the biodynamic view of Osteopathy in the Cranial Field [34]. When studying the writings of the embryologist Blechschmidt, he was impressed by Blechschmidt’s conclusion that embryonic function creates form and precedes structure [35]. Jealous hypothesized that the forces of embryologic development did not cease to function at birth but were maintained throughout our lives as forces of growth and development, and were also involved in healing processes. He describes a rhythm slower than PRM/CRI, which he simply calls the “21/2 CPM rate”, with a frequency of 2.5 cycles/min [10].

### 3.8. Entrainment Frequency

Finally, a series of articles hypothesize that the PRM would be **a result of a combination of several rhythms** that intertwine (Figure 2).

Norton [33], with his tissue pressure model, proposed that the CRI is associated with a slow adaptation of the cutaneous mechanoreceptors of both patient and practitioner, and that the sources of change in these tissue pressures are the combined respiratory and cardiovascular rhythms of both examiner and subject.

McPartland [10,14] proposes that dysfunction of the autonomic nervous system may change the entrainment frequency, causing perturbations in the PRM, altering its rate and amplitude. He suggested that the CRI is the palpable perception of entrainment, a harmonic frequency that incorporates the rhythms of multiple biological oscillators, not only heart rate and respiration, but also HRV and arterial vasomotion with THM modulation, and oscillations in lymphatic vessels, glial cells and cortical metabolism.

This model includes the examiner’s own rhythms and could therefore explain the differing rates of CRI palpated on a subject by two examiners at the same time.

More recently, Whedon and Glassey [16] proposed that the balance between the sympathetic and parasympathetic nervous systems may result in frequency entrainment of multiple biological oscillators. They suggested that the state of optimal autonomic balance is consistent with increased HRV, enhanced THM waves, and a strong palpable CRI.

### 3.9. Methodological Quality of Studies

Some of the articles included in our systematic review that investigated the existence of the PRM/CRI show important biases that were already pointed out by Guillaud [36]. For instance, regarding the article by Nelson [5], only 12 subjects out of the initial 20 are the subject of statistical treatment due to the poor quality of acquisition of the other recordings, according to the authors. As the quality criteria are not mentioned, selective sorting of the data cannot be excluded, voluntary or not. Furthermore, given the small number of subjects analyzed, the statistical analysis appears to be insufficiently detailed to conclude that it is valid. More controls are needed in the methodology used so that the palpated wave could actually be linked to the PRM.

The Sergueef study [7] used randomization, blinding, a “placebo” comparative group and statistical treatment of the data. However, the values recorded before treatment in the “manipulation” group are all higher than in the “palpation” group, which raises doubts about the comparable nature of the two groups, and the power of the study is not questioned: we do not know whether the number of subjects included (23) guarantees a statistically significant result if the treatment is truly effective.

In the Sommerfeld study [20], the risk of bias is high: the raw data is not available, which does not allow us to make calculations. The averages and differences between each of the evaluators’ measurements are not available, which does not allow for drawing a Bland and Altman graph, and the interpretation of the results is not justified. Thus, this study cannot constitute definitive proof of the intra- and inter-observer reproducibility of the assessment by palpation of a PRM.

The work presented in Guillaud [36] highlighted the weakness of certain studies in demonstrating the existence of the PRM/CRI. The report is precise, but does not address the need to search the literature for elements of understanding of the mechanisms underlying CRI/PRM, which osteopaths use extensively throughout the world.

## 4. Discussion

We found that only 28 articles in peer-reviewed English-language journals were identified by searching for the keywords: “osteopathy in the cranial field”; “cranial rhythmic impulse”; and “primary respiratory mechanism”, and that only 4 of the articles were communications in general scientific journals, showing the low visibility or research interest attributed to this subject.

The methodological rigor of the articles studied was low, with only four studies having a good methodological level, and only eight using reliable and appropriate tools for their demonstrations. This methodological weakness is not conducive to the emergence of new models.

Only 4 of the 28 studies had a type rated as good, 8 as average and the rest as poor or questionable. Regarding tools, 8 items out of 28 were rated as good, 3 as average, 3 as poor and 14 as questionable. No study had three good-level items, and only five studies had two good-level items corresponding to the type of study and tools used. Finally, 15 of the 28 studies have one or two items classified as questionable.

A correct methodological approach requires not considering the hypotheses of a phenomenon until a collection of signs or evidence imposes its existence. There are now several articles using objective measurements that correlate with the subjective perception of the PRM/CRI by osteopaths. The most recent, Peltz’s study [8], comes close to the judgment criteria required by the canons of current scientific research.

It is therefore now justified to try to understand the physiological basis of the PRM/CRI phenomenon, as has been anticipated in most publications presented in this review.

Although the quality of the studies was poor, the results of our systematic review of the literature confirm the need for a paradigm shift and for more rigorous evaluation and communication of a model that is in line with the evolution of scientific data.

While Sutherland’s model is still firmly anchored in the literature, with 20 of the 28 articles referring to at least one of the five hypotheses, the model is widely criticized and often called into question (see Table 2 and Figure 2). Our results show that 25 articles proposed working hypotheses or new explanatory models, which could be taken into consideration and compared with scientific data to see the emergence of a future consensual model.

## 5. Conclusions

Our systematic review questioned the physiological mechanisms underlying the PRM/CRI. Since Sutherland’s theory, thanks to scientific progress, the knowledge about the physiological mechanisms underlying PRM has evolved. Although Sutherland’s hypothesis is still taught, the most plausible hypothesis to explain PRM/CRI is based on the variation of the texture of the extracellular matrix and fascial network, in relation to THM oscillations and other physiological rhythms that intertwine. We will examine the various hypotheses proposed to explain this rhythm in more detail in a complementary narrative review of the literature.

## Figures and Tables

**Table 1:** Grid indicating the quality criteria imposed for each selected item.

Relevance of Journals Sum (Journal Type + Q+ IF) (Minimum = 0, Maximum = 6)	Robustness of PRM/CRI Evaluation Across Study Type		Accuracy, Reliability and Reproducibility of PRM/CRI Measurement Tools
*Journal*	*Study*	*Review*	*Measurement Tool*
0 = Red (very low level)	Case studies = Red	Review on assumptions and models = Red	No assessment of the accuracy, reliability and reproducibility of the measuring tool = Red
1–2 = Orange (low level)	Observational studies = Orange	Review and narrative review = Orange	Palpation = Orange
3–4 = Yellow (medium level)	Pilot Study = yellow	Systematic review = Yellow	Non-validated measurement tools = Yellow
5–6 = Green (high level)	Mechanistic study and randomized trial = green	N/A	Precise, reliable, reproducible and validated measurement tools = Green

## Data Availability

The data that support the findings of this study are available from the corresponding author, A.G., upon reasonable request.

## Abbreviations

CRI	Cranial rhythmic impulse
CSF	Cerebro Spinal Fluid
ECG	electrocardiogram
HF	high frequency
LF	low frequency
PRM	Primary Respiratory Mechanism or Movement
SNS	Sympathetic nervous system
THM	Traube Hering Mayer
